# Discriminative gene co-expression network analysis uncovers novel modules involved in the formation of phosphate deficiency-induced root hairs in *Arabidopsis*

**DOI:** 10.1038/srep26820

**Published:** 2016-05-25

**Authors:** Jorge E. Salazar-Henao, Wen-Dar Lin, Wolfgang Schmidt

**Affiliations:** 1 Institute of Plant and Microbial Biology, Academia Sinica, Taipei, Taiwan; 2 Biotechnology Center, National Chung-Hsing University, Taichung, Taiwan; 3 Genome and Systems Biology Degree Program, College of Life Science, National Taiwan University, Taipei, Taiwan

## Abstract

Cell fate and differentiation in the *Arabidopsis* root epidermis are genetically defined but remain plastic to environmental signals such as limited availability of inorganic phosphate (Pi). Root hairs of Pi-deficient plants are more frequent and longer than those of plants grown under Pi-replete conditions. To dissect genes involved in Pi deficiency-induced root hair morphogenesis, we constructed a co-expression network of Pi-responsive genes against a customized database that was assembled from experiments in which differentially expressed genes that encode proteins with validated functions in root hair development were over-represented. To further filter out less relevant genes, we combined this procedure with a search for common *cis*-regulatory elements in the promoters of the selected genes. In addition to well-described players and processes such as auxin signalling and modifications of primary cell walls, we discovered several novel aspects in the biology of root hairs induced by Pi deficiency, including cell cycle control, putative plastid-to-nucleus signalling, pathogen defence, reprogramming of cell wall-related carbohydrate metabolism, and chromatin remodelling. This approach allows the discovery of novel of aspects of a biological process from transcriptional profiles with high sensitivity and accuracy.

Root hairs are tip growing lateral extensions of specialized cells in the epidermis that are important for the uptake of water and mineral nutrients. In *Arabidopsis*, root epidermal cells are arranged in alternating, parallel files of root hairs (trichoblasts) and non-hair cells (atrichoblasts). The identity of epidermal cells is determined by their position relative to the underlying cortical cell layer; epidermal cells that are located over a cleft of two cortical cells (H position) develop into hair cells, whereas cells that lie over periclinal cell walls (N position) adopt the non-hair cell fate^1^,^2^,^3^. Root hair cell fate is conferred by a non-cell autonomous signal presumably generated in cortex cells by JACKDAW^4^ that is stronger in cells in the H position due to a larger contact area and higher relative abundance of the leucine-rich repeat receptor kinase SCRAMBLED (SCM), which is required for signal transduction^5^. In H cells, the cortical signal represses the expression of the MYB protein WEREWOLF (WER). Together with the WD40 repeat protein TRANSPARENT TESTA GLABRA 1 (TTG1) and the MYC transcription factors GLABRA 3 (GL3) and ENHANCER OF GLABRA 3 (EGL3), WER forms an activator complex that negatively regulates the root hair cell fate by supporting transcription of the homeodomain protein GLABRA 2 (GL2). GL2 prevents cells in the N position from entering the hair cell fate by repressing a suite of transcription factors that positively regulate root hair morphogenesis^6^. Cell identity is reinforced by cell-to-cell communication during which the R3 MYB transcription factor CAPRICE (CPC) is migrating to the H position where it competes with WER for binding to the activator complex. The CPC-GL3/EGL3-TTG complex does not support *GL2* expression and the cell surrenders to the hair fate. *GL3* and *EGL3* are preferentially expressed in H cells and move to N-positioned cells to support their differentiation into non-hair cells^7^,^8^,^9^,^10^.

Although precisely patterned, the fate of epidermal cells is not irreversible and remains responsive to external and internal cues. Movement of epidermal cells from N to H positions after laser ablations or anticlinal cell divisions after which daughter cells occupy different positions relative to the underlying cortical cells caused epidermal cells to adopt the fate that is dictated by its position, indicating that positional information and not cell lineage controls cell specification^11^. Expression of *GL2* is associated with an open chromatin structure, but perception of positional information can rapidly induce an alternative state of chromatin organization^12^.

Positional information is not only important for establishing the fate of root epidermal cells, it also determines their size^13^,^14^. Mutants that cannot perceive this information such as *scm* or *wer* form short, trichoblast-like epidermal cells in both the H and N position^14^. It appears that either the strength of the signal, its perception or the transduction of positional information to downstream targets can be modulated by environmental cues. Phosphate-deficient plants form shorter cells and more cortical cells, leading to an increase in root hair frequency per unit root length^15^,^16^. It has been suggested that Pi deficiency reduces the strength of the positional signal, leading to shorter cells and to a less stringent pattern of epidermal cell that allows the formation of hairs in ectopic positions^14^. In addition, Pi deficiency increases the growth rate and the duration of root hair elongation, resulting in significantly longer hairs^16^,^17^. Increased root hair length and density is part of a complex Pi starvation response (PSR) that comprises reprogramming of primary and secondary metabolic pathways, increased expression of genes involved in the acquisition, uptake, distribution and recycling of Pi as well as alterations in root architecture^18^,^19^. These disparate responses render dissection genes that are specifically involved in a particular aspect of the PSR difficult.

Gene regulatory networks involved in epidermal cell fate specification and morphogenesis have been inferred from transcriptional profiling approaches for standard growth conditions, reflecting genetically determined developmental programs^20^. Here, we report a co-expression-based approach to identify genes with root hair-related functions among the relatively large subset of genes that are transcriptionally regulated by Pi starvation. While most co-expression analyses are based on large, non-specific databases that comprise experiments conducted with various tissues and genotypes subjected to different experimental conditions, the current approach relies on a customized database, allowing the inference of genes that are tightly associated with trichoblast differentiation. Using this method, we identified functional modules that regulate or mediate processes critical for the phenotype typical of Pi-deficient plants by dissecting putatively orchestrated gene regulation directed by common *cis*-regulatory motifs on their promoters.

## Results and Discussion

### Identification of Pi-responsive genes related to root hair morphogenesis

To associate Pi-responsive genes with the induction of the root hair phenotype typical of Pi-deficient plants, we first normalized a set of 3,800 ATH1 microarray hybridizations collected from the NASCarray database. Microarray experiments that discriminate processes associated with root hair development were selected based on a positive gene list that comprised 56 genes with validated roles in root hair differentiation (Supplemental Table S1). We then selected microarrays in which more than 70% of the genes from this list were among the probe sets with the 25% highest or lowest signal strength. This procedure yielded 111 matches that fulfilled these criteria. Next, we selected genes that were defined as Pi-responsive at *P* < 0.05 from a previously conducted RNA-seq-based transcriptomic survey^21^. The pairwise correlation of the 1,701 Pi-responsive genes was then calculated based on this database using the in-house software package MACCU^22^, yielding five larger clusters with 120 (C0), 71 (C1), 50 (C2), 11 (C3) and 11 (C4) genes (Supplemental Table S2).

To further reduce the complexity of the network and to identify co-regulated modules that are particularly involved in altering the root hair phenotype in response to Pi deficiency, we searched for *cis*-regulatory elements (CREs) in the promoters that are significantly over-represented within these five clusters. To this end, we employed the MEME-LaB toolbox designed for *ab initio* motif finding in co-expressed gene clusters^23^. Subsequently, the motifs identified in the promoters of at least four genes were compared for similarity with known motifs of transcription factor binding sites (TFBS) using the motif database scanning algorithm Tomtom within the MEME Suite web server (http://meme-suite.org) and the regulatory sequence analysis tools (RSAT, http://rsat.ulb.ac.be/rsat/) to identify previously identified TFBS^24^,^25^. A total of 14 CREs were identified in the promoters of the genes in clusters 0 to 4 by this approach (Supplemental Table S3).

### Genes involved in cell wall organization are up-regulated by Pi deficiency

**Figure 1:**
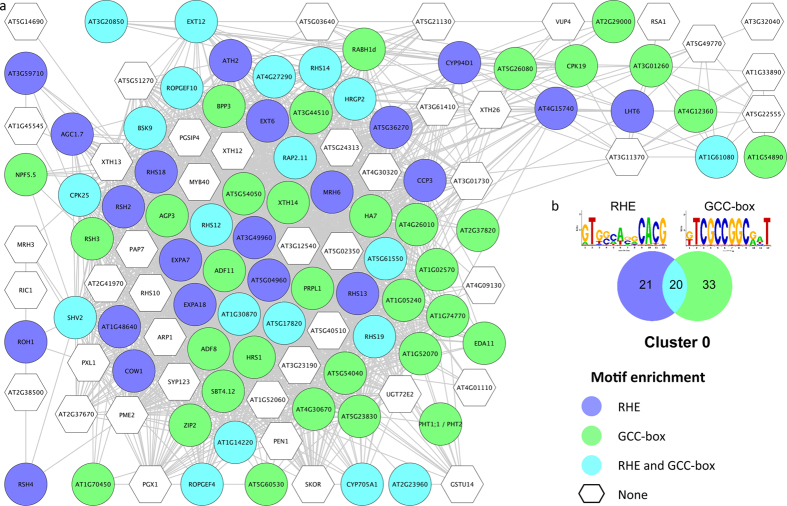
Cluster 0 of the co-expression network comprising genes that were differentially expressed in roots. (**a**) Genes were clustered based on their co-expression relationships with a Pearson’s correlation coefficient of ≥0.90. The colour of the nodes indicates the presence of either the RHE, the GCC-box or both motifs as indicated in b. (**b**) Logo, colour code and number of genes that contain the motifs.

**Figure 2:**
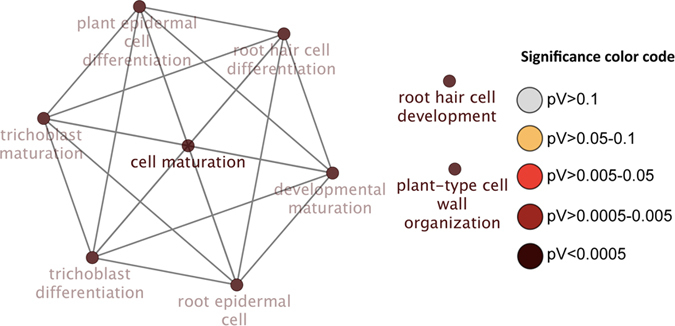
Visualization of the non-redundant biological gene ontology terms. The size of the nodes corresponds to the number of the genes associated with a term. The significance is represented by the colour of the nodes. Networks were constructed by ClueGo and displayed in ‘significance view’ by Cytoscape (http://apps.cytoscape.org/apps/cluego).

The largest cluster (C0) contains genes that are mainly related to cell morphogenesis, with the GO categories ‘cell maturation’, ‘root hair development’ and ‘cell wall organization’ strongly over-represented (Figs 1 and 2). Most of the genes from this cluster encode proteins that are predicted to localize to the extracellular space or on the plasma membrane; almost all genes were up-regulated upon Pi deficiency^21^. A subset of 56 genes was previously defined as being preferentially expressed in root hairs^26^, some of which showed more than 1,000-fold enrichment (*e.g. HRGP2*). For mutants defective in the expression of the gene encoding the unknown protein At3g49960, the peroxidase superfamily protein At1g05240, the serine protease inhibitor family protein *CCP3* and the ATPase *AHA7*, we previously reported a short root hair phenotype and reduced abundancy of root hairs, respectively under control conditions^26^. For *aha7* also a reduced frequency of root hairs under iron-deficient conditions was observed^27^. Mutants defective in the expression of *ROPGEF4* and At3g01730 were reported to form longer (*ropgef4*) or shorter (At3g01730) root hairs under Pi-deficient conditions when compared to wild-type plants^22^.

### Genes that define the length and shape of root hair cells of Pi-deficient plants are co-expressed with genes encoding cell wall-modifying proteins

The GARP transcription factor HRS1 HOMOLOGUE1 (HHO) is a Pi and nitrate signal integrator that represses primary root growth in the absence of Pi^28^. *HHO* is expressed in nuclei of elongating root cells and was up-regulated by Pi deficiency in our survey^21^,^28^. In contrast, POLYGALACTURONASE INVOLVED IN EXPANSION1 (PGX1) promotes cell elongation and is expressed in tissues undergoing cell expansion, including root tips^29^. *PGX1* was down-regulated upon Pi starvation and may be involved in the regulation of the attenuation of root cell elongation upon Pi deficiency.

Expression of the proline-rich protein-like *PRPL1* gene is restricted to trichoblasts^26^ and the protein was functionally associated with the elongation of root hairs^30^. Upon Pi starvation, the transcript level of *PRPL1* was increased approximately 2-fold^21^. Thus, in conjunction with proteins involved in cell wall organization, induction of the proteins encoded by these genes might be involved in orchestrating the Pi deficiency root hair phenotype.

### Root hair morphogenesis under Pi-deficient conditions is regulated by two major CREs

In cluster 0, the Root Hair Element (RHE^31^,^32^) was identified in the promoters of 42 genes. Two cysteine/histidine-rich C1 domain family proteins (At5g54050 and At5g54040), annotated as being involved in intracellular signal transduction, are up-regulated upon Pi deficiency with At5g54040 having substantially (more than 20-fold) higher expression levels^21^. The role of the two proteins in Pi deficiency-induced root hair formation is unclear at present. In eukaryotes, the C1 domain can bind diacylglycerol (DAG), which acts as a second messenger in animals. In plants, DAG is rapidly converted to phosphatidic acid (PA) by DAG kinase, which is more likely to act as a signalling molecule in plants^33^,^34^. Under Pi-deficient conditions, PA and DAG participate in membrane lipid remodelling, a pathway in which phospholipids are substituted by the galactolipid digalactosyldiacylglycerol (DGDG) and the sulfolipid sulfoquinovosyldiacylglycerol^35^,^36^. Lipid metabolism under Pi deficiency has been implicated in root hair development^37^,^38^, supporting the concept of tightly intertwined metabolic and developmental Pi starvation responses. Co-regulation of At5g54050 and At5g54040 with genes involved in root hair morphogenesis provides a further link of lipid metabolism and/or signalling with the root hair phenotype of Pi-deficient plants.

A second consensus motif, the GCC box (GCCGNM), was present in the promoters of 55 genes, among them the BRASSINOSTEROID-SIGNALING KINASE 9 (BSK9). BSKs have been shown to be part of the brassinosteroid (BR) signalling pathway down-stream of BRI1^39^. *BSK9* is co-expressed with several key genes in root hair development that are highly enriched in trichoblasts, but *BSK9* itself is not preferentially expressed in root hairs^26^. Compromised BR signalling results in impaired expression of *CPC* and, consequently, in a decrease of hair formation in the H position^40^. Brassinosteroids have been further implicated in a mechanism that involves protein phosphorylation of the cell specification proteins EGL3 and TTG1 via the GSK3-like kinase BIN2, leading to inhibition of the WER-GL3/EGL3-TTG1 activator complex^41^. *BSK9* was strongly up-regulated by Pi deficiency. Interestingly, the plasma membrane-bound Pi transporters PHT1;1 and PHT2 contain the GCC box in their promoters, suggesting a close co-regulation of genes involved in root hair elongation and Pi uptake.

Several AP/EREBP transcription factors have been identified as regulatory proteins that can interact with the GCC-box^42^,^43^,^44^,^45^. The AP2/ERF protein RAP2.11 is a putative *trans*-acting factor for genes harbouring the GCC-box in cluster 0. RAP2.11 is co-expressed with several genes with highly specific expression in root hairs such as *EXP7* and *EXP18*, as well as some *ROOT HAIR SPECIFIC* (*RHS*) genes that contain the RHE consensus. Consistent with a role in nutrient signalling, *RAP2.11* is preferentially expressed in root epidermal cells and in the root cap^46^. *RAP2.11* over-expression lines form shorter primary roots and a more numerous root hairs, resembling Pi-deficient plants. Moreover, the H_2_O_2_ producing class III peroxidase *PRX34* was down-regulated in *rap2.11* plants^46^, indicative of a putative role of RAP2.11 in ROS homeostasis. *PRX34* expression was also strongly decreased in mutants defective in the expression of the mediator subunit *PFT1/MED25*^47^. PFT1/MED25 is critical for root hair morphogenesis and *pft1* mutants showed perturbed ROS distribution along roots, indicating a strong linkage of root hair differentiation and redox control via class III peroxidases.

Notably, 31 of the genes in cluster 0 are regulated by the bHLH transcription factor ROOT HAIR DEFECTIVE 6-LIKE 4 (RSL4), a direct target of ROOT HAIR DEFECTIVE 6 (RHD6) which positively regulates the root hair cell fate^48^,^49^. RSL4 is sufficient to initiate root hair growth and is up-regulated by Pi deficiency. It has been suggested that RSL4 integrates developmental and environmental signals to define the length of the hairs^49^.

### A role for plastid-to-nucleus signalling in the regulation of PSR genes

**Figure 3:**
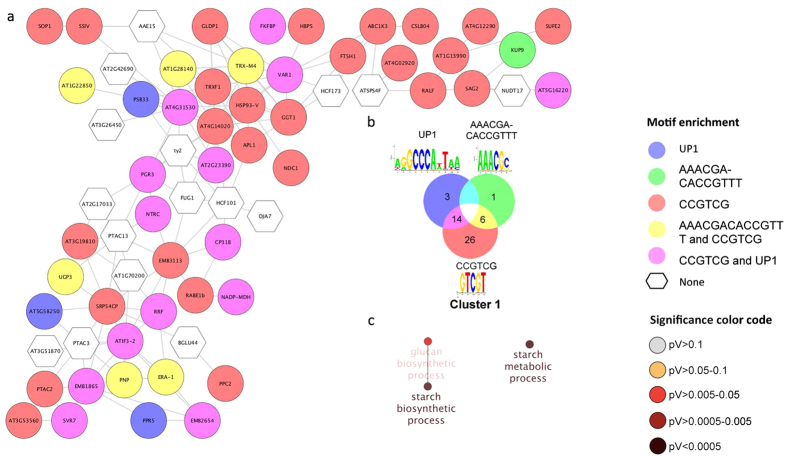
Cluster 1 of the co-expression network comprising genes that were differentially expressed in roots. (**a**) Genes were clustered based on their co-expression relationships with a Pearson’s correlation coefficient of ≥0.90. The colour of the nodes indicates the presence of the various motifs indicated in b. (**b**) Logo, colour code and number of genes that contain the motifs. (**c**) Visualization the non-redundant biological gene ontology terms. The size of the nodes corresponds to the number of the genes associated to a term. The significance is represented by the colour of the nodes. Networks were constructed by ClueGo and displayed in ‘significance view’ by Cytoscape (http://apps.cytoscape.org/apps/cluego).

Genes in cluster 1 are associated with the regulation of carbohydrate metabolic processes, in particular the biosynthesis of starch and glucan (Fig. 3). The majority of genes in this cluster (54 out of 72) encode proteins that are predicted to localize to plastids, indicating a possible connection between plastid metabolism and Pi deficiency-induced root hair formation. Pi starvation strongly compromises photosynthesis (PS) and regulatory (nucleic) nodes of the Pi starvation responses could be coupled to the down-regulation of PS-related genes. Such a regulatory circuit might be conserved among cells of different tissues independent on their photosynthetic activity. Cluster 1 contains several genes encoding proteins with RNA-binding and/or pentatricopeptide repeat (PPR) motifs (*PGR3*, *SVR7*, At2g17033, At1g70200, *ERA-1*, *CP31B*), transcriptional regulators (*PTAC2*, *PTAC3*, *PTAC13*, *EMB1856*) and translation initiation/elongation factors (*SCO1*, *FUG1*, *IF3-2*), which are mostly down-regulated in Pi-deficient plants. PPR-motif containing proteins such as PGR3 have been shown to stabilize RNA and to activate translation, thereby modulating plastid gene activity^50^. Also, several genes involved in carbohydrate metabolism are in this cluster.

The chloroplast polynucleotide phosphorylase (PNPase) PNP/RIF10 has been associated with the Pi starvation response of roots. *pnp* plants form fewer lateral roots in response to Pi starvation and up-regulate a large subset of Pi starvation genes when grown on Pi-replete media, among them genes related to root hair formation specifically under Pi deficient conditions such as *WRKY75*^51^,^52^. Most interestingly, a *Chlamydomonas reinhardtii* mutant with compromised *PNP* expression was unable to induce Pi starvation responses, indicating conserved function of PNP in unicellular and multicellular organisms^51^. In our RNA-seq survey, *PNP* was down-regulated upon Pi deficiency^21^. As pointed out by Marchive *et al.*^51^, a plausible scenario could involve sugar signalling that is related to the nutritional state of plastids that, in interaction with Pi-responsive proteins, triggers the induction of systemic Pi starvation signals.

### Promoter motif analysis supports a role of plastid-located proteins in cellular Pi homoeostasis

The motif AAACGACACCGTTT was found in the promoters of genes encoding PNP, the unknown protein At1g28140, the tetratricopeptide repeat (TPR)-like superfamily protein At3g53560, ERA-1 and a SNARE-associated Golgi protein family protein (At1g22850) which is tightly co-expressed with the chloroplast-located low affinity Pi transporter *PHT2.1* (Fig. 3). At1g22850 is annotated as being involved in the biosynthesis of myo-inositol hexakisphosphate (InsP6), a storage form of Pi. Interestingly, inositol polyphosphate kinases have been implicated in root hair elongation and Pi sensing^53^. Regulation of genes in this module may affect root hair elongation either directly via signalling molecules or indirectly via alterations of the plant’s Pi status.

The promoters of *PNP*, At1g22850 and At1g28140 also contained the motif CCGTCG, shared with 49 genes in this cluster that are mainly related to carbohydrate metabolism, plastid organization and translation. PROTON GRADIENT REGULATION 3 (PGR3) has been associated with stabilization of *photosynthetic electron transport L* (*petL*) operon mRNA^54^, thereby supporting translation of the encoded proteins. Most genes in this sub-cluster were down-regulated under Pi-deficient conditions, suggesting decreased activity of genes encoding proteins that localize to plastids during Pi starvation. The consensus motif UP1, which was found to be enriched in the promoters of genes that were up-regulated after the decapitation of the main stem in *Arabidopsis*^55^, was present in 18 genes, which are enriched in the GO category ‘embryonic development’. A subset of six genes contained both CCGTCG and AAACGACACCGTTT, among them *PNP*, At1g28140, At1g22850 and *ERA-1*, indicative of tight co-regulation of these genes. The promoters of 15 genes, which include *PGR3*, contain both CCGTCG and UP1.

### Chromatin conformation dynamics modulates root hair formation in response to Pi deficiency

**Figure 4:**
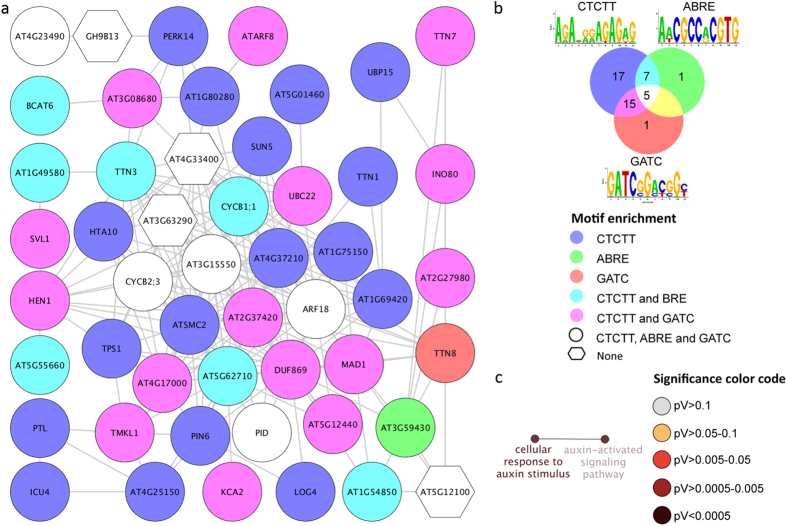
Cluster 2 of the co-expression network comprising genes that were differentially expressed in roots. (**a**) Genes were clustered based on their co-expression relationships with a Pearson’s correlation coefficient of ≥0.90. The colour of the nodes indicates the presence of the various motifs indicated in b. (**b**) Logo, colour code and number of genes that contain the motifs. Network is visualized by Cytoscape 3.2.1 (http://www.cytoscape.org). (**c**) Visualization the non-redundant biological gene ontology terms. The size of the nodes corresponds to the number of the genes associated to a term. The significance is represented by the colour of the nodes. Networks were constructed by ClueGo and displayed in ‘significance view’ by Cytoscape (http://apps.cytoscape.org/apps/cluego).

In cluster 2, genes that relate to the GO categories ‘cellular response to auxin stimulus’ and ‘auxin-activated signalling pathway’ are over-represented (Fig. 4). Most proteins encoded by genes in this cluster encode proteins that are predicted to localize to the nucleus. Genes related to cell division (*i.e. TTN1*, *TTN3*, *TTN7*, *TTN8*, *MAD1*, *SCMC2*, *CYCB2;3*, *CYCB1;1*, At1g20590) were all down-regulated by Pi deficiency. Also, several genes related to chromatin silencing (*INO80*, *HEN1*, *TTN3*, *KAC2*, At1g75150, and At2g27980) had decreased abundance in Pi-deficient plants, while the gene encoding the histone H2A variant HTA10 was up-regulated. These changes are indicative of dynamic restructuring of chromatin in response to Pi starvation. INO80 has been detected in root hair-specific proteomic analysis as being preferentially expressed in trichoblasts^26^, indicating robust presence of INO80 in root hairs both at the transcript and protein level. INO80 encodes a conserved chromatin-remodelling complex^56^,^57^ that interacts with H2A.Z. Proper deposition of H2A.Z is required for PSR gene induction^58^. The *arp6* (*actin-related protein 6*) mutant is defective in a nuclear actin-related component of the chromatin remodelled SWR1^59^, resulting in perturbed deposition of H2A.Z and constitutive formation of long and dense root hairs reminiscent of Pi-deficient plants^58^. It might thus be speculated that eviction of the canonical histones H2A/H2B is coupled to the deposition of H2A.Z and HDA10 in response to Pi starvation and may be required for proper regulation of a subset of PSR genes.

### An auxin-induced regulatory module links chromatin dynamics to root hair development

The kinase PINOID (PID), a positive key regulator of polar auxin transport and a negative regulator of root hair growth^60^, was down-regulated upon Pi deficiency^21^. Proper *PID* expression was shown to be dependent on the expression of a neighbouring transcript, the long intergenic non-coding RNA (lincRNA) *APOLO*, which is transcribed by both Pol II and Pol V^61^. This dual expression induces a chromatin loop that encompasses the promoter of *PID* and is controlled by DNA methylation. The dynamics of loop formation results in an oscillatory gene expression pattern, which resembles similar oscillatory patterns that determines the formation of lateral roots^62^. Both root hair formation and lateral root development are modulated by auxin dynamics and are responsive to the Pi supply; altered *PID* expression result in changes in root development^63^. It is thus tempting to speculate that such auxin-based oscillatory gene expression patterns represent important adaptive mechanisms that alter developmental programs in response to environmental cues. Interestingly, RNAi *APOLO* plants with compromised *PID* expression form longer roots, a phenotype that is opposite to that of Pi-deficient plants^61^.

All but two genes in this cluster carry the CRE CCGTCG in their promoters, suggesting tight co-regulation of the genes in this module. A second CRE (TGATCR) was found in 22 genes, and a subset of 20 genes, including *INO80*, *HEN1*, the auxin response factors *ARF8* and *ARF18*, as well as *PID*, carry both DNA motifs in their promoters. This module also contains the glycerophosphoryl diester phosphodiesterase-like protein *GDPDL4/SVL3*. *SVL3* is an allele of the *shaven* mutant in which root hairs are ruptured during tip growth due to impaired primary cell wall organization^64^,^65^. *GDPDL4* links this module to root hair development.

A third CRE (CACGTGGC) is present in the promoters of 13 genes. A small subset of genes contains all three motifs (*PID*, *ARF18*, *CYCB2;3* and two genes encoding unknown proteins, At4g23490 and At3g15550). At4g23490 is co-expressed with genes involved in cell wall-related processes. For At3g15550, BLAST results suggest similarity with chromatin remodelling proteins.

### Genes involved in secondary cell wall formation are down-regulated upon Pi deficiency

**Figure 5:**
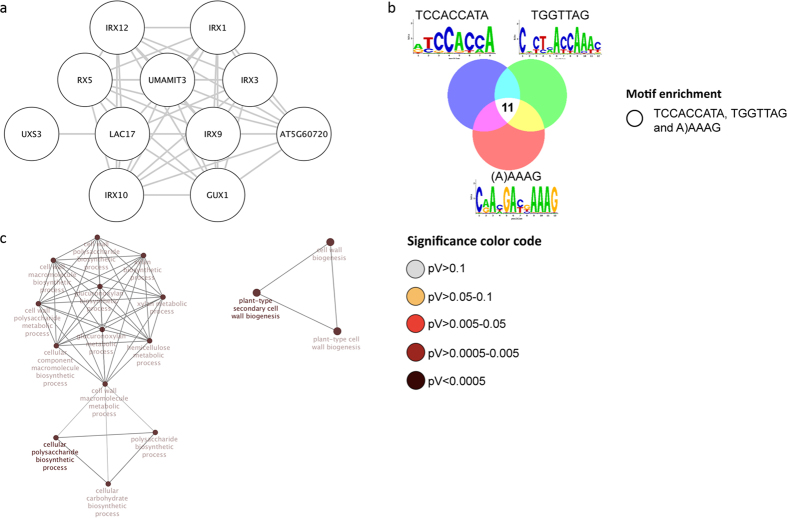
Cluster 3 of the co-expression network comprising genes that were differentially expressed in roots. (**a**) Genes were clustered based on their co-expression relationships with a Pearson’s correlation coefficient of ≥0.90. Nodes containing all three motifs indicated in b. (**b**) Logo, colour code and number of genes that contain the motifs. (**c**) Visualization the non-redundant biological gene ontology terms. The size of the nodes corresponds to the number of the genes associated to a term. The significance is represented by the color of the nodes. Networks were constructed by ClueGo and displayed in ‘significance view’ by Cytoscape (http://apps.cytoscape.org/apps/cluego).

Cluster 3 contains genes that are related to the biosynthesis of polysaccharides and secondary cell walls (Fig. 5). Several *IRREGULAR XYLEM* (*IRX*) genes and the xylan glucuronyl transferase *GUX1* are part of this cluster. GUX1 is involved in the addition of glucuronic acid and methylglucuronic acid residues onto xylan and for secondary wall deposition^66^,^67^. Another gene in this cluster, *UXS3*, produces UDP-xylose, a sugar donor for many cell wall carbohydrates such as hemicellulose and pectin. *LAC17* encodes a protein that controls lipid deposition during protoxylem tracheary element development^68^, an unknown gene, At5g60720, has been associated with secondary wall formation in transcriptomic profiling experiments^69^,^70^. All genes were down-regulated upon Pi starvation and all genes contain three different CREs, ACCACCAAA, TGATTAG and AAAAG.

### Glucosinolate biosynthesis genes are up-regulated upon Pi deficiency

**Figure 6:**
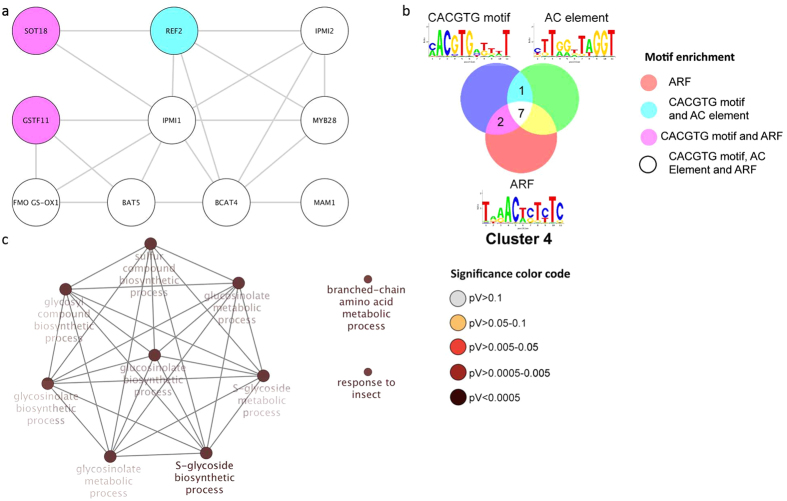
Cluster 4 of the co-expression network comprising genes that were differentially expressed in roots. (**a**) Genes were clustered based on their co-expression relationships with a Pearson’s correlation coefficient of ≥0.90. Nodes contain all three motifs indicated in b. (**b**) Logo, colour code and number of genes that contain the motifs. (**c**) Visualization the non-redundant biological gene ontology terms. The size of the nodes corresponds to the number of the genes associated to a term. The significance is represented by the colour of the nodes. Networks were constructed by ClueGo and displayed in ‘significance view’ by Cytoscape (http://apps.cytoscape.org/apps/cluego).

Cluster 4 contains genes related to methionine-derived glucosinolate biosynthesis: *MAM1*, *IPMI1*, *IPMI2*, *IMD1*, *BAT5*, *GSTF11*, *BCAT4*, *REF2*, *SOT18*, *FMO GS-OX1* and *MYB28* (Fig. 6). The genes in this cluster appear to be tightly co-regulated, indicated by the presence of three different motifs in the promoters of most of the genes. Ten genes carry the CACGTG motif and the AC element, all three CREs together are found in the promoters of seven genes. Unsurprisingly, the GO categories ‘S-glycoside biosynthetic process’, ‘branched-chain amino acid metabolic process’ and ‘response to insect’ are strongly over-represented in this cluster (Fig. 6).

## Conclusions

The present study describes a feasible approach to filter gene expression profiles with the aim of discovering novel aspects of a biological process. We reckon that with this approach relatively subtle but nevertheless critical changes in transcript abundance can be separated from stochastic noise that is inherent with transcription. It can be further stated that the identification of such aspects is strongly facilitated by the RNA-seq technology that allows the detection of robust changes in gene expression independent of a set threshold that is often used for microarray analyses because of high fluctuations among replicate experiments caused by the relatively low sensitivity of the probe sets. Our approach captured a large subset of cell wall-modulating genes (cluster 0) that were previously shown to be critical for proper root hair development. These data suggest that many genes that mediate critical functions in root hair differentiation under control (Pi-replete) conditions are also important for the induction of the elongated root hairs of Pi-deficient plants. It can further be stated that transcriptional up-regulation of these genes is most likely causally linked to the phenotype of Pi-deficient plants.

A further novel aspect is the putative involvement of plastids in the root PSR response. Plastids are critical players in sensing environmental cues and efficient communicators of environmental conditions^71^. Metabolic changes in plastids can affect the plant’s overall transcript profiles and plastid-to-nucleus retrograde signalling provides a means to relay environmental signals to regulate the expression of nuclear genes^72^. While signalling between chloroplasts and the nucleus is generally associated with photosynthetic active cells, recent evidence indicates that signals perceived in chloroplasts might also affect root processes. A recent study showed that light-induced alternative splicing triggered by metabolic changes in chloroplasts was only observed in roots when their connection to the leaves was intact, suggesting that the induction of alternative splicing in roots requires a signal that travels form shoots to roots^73^. Retrograde signalling can be initiated by changes in the oxidation state of plastoquinone, which is likely to be affected by Pi deficiency as well. Three possible, not mutually exclusive scenarios could explain how the low Pi status of plastids is conveyed to the nucleus. Firstly, changes in the plastid metabolic state might trigger the production of signalling molecules that exit the organelle and are transported into the nucleus where they induce the expression of PSR genes. This scenario is supported by the observation that the *hypersensitive to Pi starvation7* (*hps7*) mutant exhibited a more severe attenuation of root growth when compared to the wild type^74^. Root growth inhibition in this mutant was associated with an up-regulation of photosynthesis-related genes and subsequent accumulation of ROS in roots that may trigger the expression of growth-related genes. The H_2_O_2_ concentration decreases in response to Pi deficiency, which has been linked to morphological alterations caused by Pi deficiency^75^. Secondly, increased transport and sequestering of Pi in the plastid may deplete cytoplasmic Pi pools and indirectly trigger the expression of nuclear PSR genes. In an alternative scenario, reduced PS and altered states of photosynthetically active chloroplasts in the leaves might induce the production of a leaf-to-root signal that communicates the leaf’s Pi status to the roots and initiate the root PSR. In this case, alterations in root plastid gene expression are conserved and mirror the responses of leaves without having direct effects on the root PSR. Such plastid-to-nucleus signalling might also be important for other nutrient deficiencies that have a strong impact on plastid-related processes such as iron or manganese deficiency.

An unexpected finding of our investigation was the possible involvement of chromatin organization in the PSR. Our data suggest that chromatin loop dynamics are involved in the formation of root hairs in response to Pi deficiency-triggered auxin stimuli in a scenario in which increased auxin responsiveness upon Pi deficiency and active DNA demethylation may fine-tune *PID* and *ARF* expression to increase root hair density of Pi-deficient plants. DNA methylation patterns were shown to be of critical importance for a proper Pi starvation response^76^. Loop formation appears to be required for proper auxin signaling in response to Pi deficiency, which in turn is critical for the root hair phenotype typical of Pi-deficient plants.

We also observed down-regulation of genes involved in secondary cell wall formation as part of a reprogramming of cell wall–related carbohydrate metabolisms under conditions of Pi deficiency and an up-regulation of genes involved in glucosinolate biosynthesis. Glucosinolates are chemo-protective secondary metabolites mainly found in the order Brassicales that have repellent activity against insects and pathogens. Plant responses to pathogens are thought to be organ-specific, but only a few studies compared the resistance mechanism of different plant parts^77^. Both leaves and roots are capable to synthesize methionine-derived aliphatic and tryptophan-derived indolic glucosinolates^78^, but roots contain more indolic glucosinolates compared to leaves^79^. Both aliphatic and indolic glucosinolates are produced in *Arabidopsis* trichomes to protect the plant against pathogens^80^. Similar to trichomes, root hairs represent the first barrier to the environment, but knowledge about defence responses in roots is limited. Secreted secondary metabolites are critical in root resistance responses^81^ but glucosinolates have not been implicated with root hairs. In the present study, we observed robust up-regulation of genes involved in alipathic glucosinolate production in roots upon Pi deficiency in a context that discriminates for processes related to root hair development. This is indicative of an increased defence requirement under low Pi conditions. Glucosinolate biosynthesis has been mainly associated with above-ground defence responses; the observed up-regulation of glucosinolate biosynthesis was unanticipated and adds a novel aspect to the PSR of *Arabidopsis*.

In summary, the current data allow for the discovery of novel aspects of root hair development in response to Pi starvation that can guide follow-up research, to validate and extent the present findings. It should be noted that while our data have been filtered for genes involved in root hair morphogenesis, the biological processes described here may also reflect responses to Pi starvation that are not directly related to root hair formation or function. Although the transcriptional response to Pi deficiency is well explored, the identification of genes that are critical for a particular aspect of the acclamatory response to Pi shortage among hundreds or thousands of genes with altered expression is a challenging task. The current approach is suitable to identify functional modules comprising genes with relatively subtle changes in transcript abundance in response to changing Pi supply. The method described here can be used to analyse RNA-seq data for a more target-oriented dissection of transcriptional changes of high fidelity.

## Methods

### Co-expression analysis

Discriminative co-expression analysis was performed as described in Lan *et al.*^26^ with the MACCU software (http://maccu.sourceforge.net/) based on co-expression relationships with a Pearson’s coefficient greater than or equal to 0.90. Root hair-related microarray experiments were selected based on a positive gene list comprising genes with validated function in root development (Supplemental Table 1) and downloaded from NASCArrays (http://affymetrix.arabidopsis.info/). Visualization of the networks was performed with the Cytoscape software version 3.2.0 (http://www.cytoscape.org/).

### Promoter motif analysis

The MEME algorithm available with the web tool MEME-LaB, accessible at http://wsbc.warwick. ac.uk/wsbcToolsWebpage/23,82, was used for discovery of promoter motifs. The parameters used in MEME-LaB were: promoter max length: 1000; stop at neighbouring gene for core promoter: TRUE; promoter min length: 50; use repeat masked sequences: TRUE; number of motifs: 10; min motif width: 6; max motif width; 12; include background model: Markov Chain Order 3 from RSAT pre-calculated background models (http://floresta.eead.csic.es/rsat/). The parameters used for background generation were: Markov order: 3; organism: Arabidopsis; sequence type: upstream; count on: both strands; prevent overlapping matches (noov); pseudo-frequencies: 0; output format: meme; decimals: 4. Subsequently, the identified motifs were compared with known TFBS using the web tools MEME-LaB and Tomtom^25^. Tomtom is part of the MEME Suite online platform (http://meme-suite.org/index.html). The resulting TFBS models were then manually curated to select the best model with relation to experimental data. In addition, the DNA-pattern software available at http://floresta.eead.csic.es/rsat/ was used for the identification of the TFBS in the promoter regions of the genes in each cluster.

### GO Analysis

GO enrichment analysis of genes sets was performed using the ClueGO^83^ version 2.0.7 plugin tool in Cytoscape^84^ version 3.2.1 with the GO Biological Process category. Overrepresented Biological Process categories were identified using an (right-sided) enrichment test based on the hypergeometric distribution. To correct the P-values for multiple testing Bonferroni step-down was used.

## Additional Information

**How to cite this article**: Salazar-Henao, J. E. *et al.* Discriminative gene co-expression network analysis uncovers novel modules involved in the formation of phosphate deficiency-induced root hairs in *Arabidopsis. Sci. Rep.* **6**, 26820; doi: 10.1038/srep26820 (2016).

## Supplementary Material

Supplementary Information

Supplementary Table S2

Supplementary Table S3
