# Supplementary material for: Discriminative gene co-expression network analysis uncovers novel modules involved in the formation of phosphate deficiency-induced root hairs in Arabidopsis
Source: Sci Rep. 2016 May 25;6:26820. doi: 10.1038/srep26820 (PMC4879556; doi:10.1038/srep26820)
Supplement: Supplementary Information [file srep26820-s1.doc]

**ONLINE DATA SUPPLEMENT**

**Discriminative gene co-expression network analysis uncovers novel modules involved in the formation of phosphate deficiency-induced root hairs in *Arabidopsis***

**Jorge E. Salazar Henao^1^, Wen-Dar Lin^1^ and Wolfgang Schmidt^1,2,3*^**

^1^Institute of Plant and Microbial Biology, Academia Sinica, Taipei, Taiwan

^2^Biotechnology Center, National Chung-Hsing University, Taichung, Taiwan

^3^Genome and Systems Biology Degree Program, College of Life Science, National Taiwan University, Taipei, Taiwan

^*^corresponding author

Correspondence to [wosh@gate.sinica.edu.tw](mailto:wosh@gate.sinica.edu.tw)

**Supplementary Table 1.** Positive genes used for the search for expression signatures among publicly available microarray hybridizations.

**Supplementary Table 2.** Genes in clusters 0 to 4.

**Supplementary Table 3.** Identified *cis*-regulatory motifs in the promoters of the genes in clusters 0 to 4.

**Supplemental Table 1.** Positive genes used for the search for expression signatures among publically available microarray hybridizations.

| Locus | Name |
| --- | --- |
| [At1g54970](http://www.arabidopsis.org/servlets/TairObject?id=27342&type=locus) | PRP1 (RHS7) |
| [At3g62680](http://www.arabidopsis.org/servlets/TairObject?id=36883&type=locus) | PRP3 |
| [At3G54590](http://www.arabidopsis.org/servlets/TairObject?id=39548&type=locus) | HRGP1 |
| [At4g25820](http://www.arabidopsis.org/servlets/TairObject?id=126954&type=locus) | XTH14 |
| At1g12040 | LRX1 |
| [At1g62440](http://www.arabidopsis.org/servlets/TairObject?id=137516&type=locus) | LRX2 |
| [At1g12560](http://www.arabidopsis.org/servlets/TairObject?id=30618&type=locus) | EXP7 |
| [At1g62980](http://www.arabidopsis.org/servlets/TairObject?id=28051&type=locus) | EXP18 |
| [At1g05990](http://www.arabidopsis.org/servlets/TairObject?id=136829&type=locus) | RHS1 |
| [At1g34760](http://www.arabidopsis.org/servlets/TairObject?id=26877&type=locus) | RHS5 (GRF11) |
| [At1g63450](http://www.arabidopsis.org/servlets/TairObject?id=30091&type=locus) | RHS8 |
| [At1g69240](http://www.arabidopsis.org/servlets/TairObject?id=29316&type=locus) | RHS9 |
| [At1g70460](http://www.arabidopsis.org/servlets/TairObject?id=29390&type=locus) | RHS10 |
| [At2g45890](http://www.arabidopsis.org/servlets/TairObject?id=33076&type=locus) | ROPGEF4 (RHS11) |
| [At3g10710](http://www.arabidopsis.org/servlets/TairObject?id=40634&type=locus) | RHS12 |
| [At4g02270](http://www.arabidopsis.org/servlets/TairObject?id=129842&type=locus) | RHS13 |
| [At4g22080](http://www.arabidopsis.org/servlets/TairObject?id=127388&type=locus) | RHS14 |
| [At4g25220](http://www.arabidopsis.org/servlets/TairObject?id=127694&type=locus) | RHS15 |
| [At4g29180](http://www.arabidopsis.org/servlets/TairObject?id=127296&type=locus) | RHS16 |
| [At4g38390](http://www.arabidopsis.org/servlets/TairObject?id=127566&type=locus) | RHS17 |
| [At5g22410](http://www.arabidopsis.org/servlets/TairObject?id=134979&type=locus) | RHS18 |
| [At5g67400](http://www.arabidopsis.org/servlets/TairObject?id=132482&type=locus) | RHS19 |
| [At1g75840](http://www.arabidopsis.org/servlets/TairObject?id=137559&type=locus) | ROP4 |
| [At4g35020](http://www.arabidopsis.org/servlets/TairObject?id=129014&type=locus) | ROP6 |
| [At1g20090](http://www.arabidopsis.org/servlets/TairObject?id=136787&type=locus) | ROP2 |
| [At4g23640](http://www.arabidopsis.org/servlets/TairObject?id=128503&type=locus) | TRH1 |
| [At1g66470](http://www.arabidopsis.org/servlets/TairObject?id=29754&type=locus) | RHD6 |
| [At1g27740](http://www.arabidopsis.org/servlets/TairObject?id=136883&type=locus) | RSL4 |
| [At1g24180](http://www.arabidopsis.org/servlets/TairObject?id=30251&type=locus) | IAR4 |
| [At4g33880](http://www.arabidopsis.org/servlets/TairObject?id=127155&type=locus) | RSL2 |
| [At5g51060](http://www.arabidopsis.org/servlets/TairObject?id=132363&type=locus) | RHD2 |
| [At5g41315](http://www.arabidopsis.org/servlets/TairObject?id=500229739&type=locus) | GL3 |
| [At2g20520](http://www.arabidopsis.org/servlets/TairObject?id=33736&type=locus) | FLA6 |
| [At4g25820](http://www.arabidopsis.org/servlets/TairObject?id=126954&type=locus) | XTH14 |
| [At4g28850](http://www.arabidopsis.org/servlets/TairObject?id=127008&type=locus) | XTH26 |
| [At3g12110](http://www.arabidopsis.org/servlets/TairObject?id=39963&type=locus) | ACT11 |
| [At2g26420](http://www.arabidopsis.org/servlets/TairObject?id=35562&type=locus) | PIP5K3 |
| [At1g48380](http://www.arabidopsis.org/servlets/TairObject?id=26768&type=locus) | RHL1 |
| [At5g49270](http://www.arabidopsis.org/servlets/TairObject?id=132170&type=locus) | SHV2/COBL9 |
| [At4g34580](http://www.arabidopsis.org/servlets/TairObject?id=130049&type=locus) | COW1/CSLD3 |
| [At3g60330](http://www.arabidopsis.org/servlets/TairObject?id=36920&type=locus) | AHA7 |
| [At4g26320](http://www.arabidopsis.org/servlets/TairObject?id=129718&type=locus) | AGP13 |
| [At3g57690](http://www.arabidopsis.org/servlets/TairObject?id=36000&type=locus) | AGP23 |
| At5g43230 | Unknown protein |
| [At2g03720](http://www.arabidopsis.org/servlets/TairObject?id=32210&type=locus) | MRH6 |
| [At3g54870](http://www.arabidopsis.org/servlets/TairObject?id=37058&type=locus) | MRH2 |
| [At4g18640](http://www.arabidopsis.org/servlets/TairObject?id=127892&type=locus) | MRH1 |
| [At4g26690](http://www.arabidopsis.org/servlets/TairObject?id=126796&type=locus) | MRH5/SHV3 |
| [At5g65090](http://www.arabidopsis.org/servlets/TairObject?id=134324&type=locus) | MRH3/DER4 |
| At3g43960 | putative cysteine proteinase |
| At4g15290 | CSLB5 |
| At3g01730 | Unknown |
| At5g51310 | 2OG family protein |
| At1g24620 | EF hand family protein |
| At2g45890 | ROPGEF4 |
| At5g56540 | AGP14 |
| At5g60660 | PIP2;4 |
